# The PPD-ACT app in Canada: feasibility and a latent class analysis of participants with postpartum depression recruited to a psychiatric genetics study using a mobile application

**DOI:** 10.1186/s12888-022-04363-7

**Published:** 2022-11-24

**Authors:** Joanna Collaton, Cindy-Lee Dennis, Valerie H. Taylor, Sophie Grigoriadis, Tim F. Oberlander, Benicio N. Frey, Ryan Van Lieshout, Jerry Guintivano, Samantha Meltzer-Brody, James L. Kennedy, Simone N. Vigod

**Affiliations:** 1 Dalla Lana School of Public Health, University of Toronto, Toronto, ON Canada; 2 Women’s College Hospital and Research Institute, 76 Grenville Street, Toronto, ON Canada; 3 Lawrence S. Bloomberg Faculty of Nursing, University of Toronto, Toronto, ON Canada; 4 Department of Psychiatry, Faculty of Medicine, University of Toronto, Toronto, ON Canada; 5 Sunnybrook Health Sciences Centre, Toronto, ON Canada; 6 BC Women’s Hospital and Health Centre, University of British Columbia, Vancouver, BC Canada; 7 Department of Psychiatry and Behavioural Neurosciences, McMaster University, Hamilton, ON Canada; 8 Women’s Health Concerns Clinic, St. Joseph’s Healthcare Hamilton ON, Hamilton, ON L8N 4A6 Canada; 9 University of North Carolina, Chapel Hill, NC USA; 10 Centre for Addiction and Mental Health, Toronto, ON Canada

**Keywords:** Post-partum depression, Feasibility, Mobile app, Study recruitment

## Abstract

**Background:**

Postpartum depression (PPD) and postpartum psychosis (PPP) are linked to negative consequences for women and families. Virtual applications present a solution to the challenge of recruiting large samples for genetic PPD/PPP research. This study aimed to evaluate the feasibility of a protocol for enrolling Canadian women with PPD and PPP to a large international psychiatric genetics study using a mobile application (PPD-ACT), and identify clinically distinct subtypes of PPD in the recruited sample.

**Methods:**

From April 2017–June 2019, Canadian women provided phenotypic data through the PPD-ACT app. Requests for a genetic sample were made from those with a current or past PPD episode based on an Edinburgh Postnatal Depression Scale (EPDS) score > 12 with onset in pregnancy or 0–3 months postpartum, and from those self-reporting lifetime PPP. Latent class analysis (LCA) was used to identify clinically distinct PPD subgroups based on participant responses to the EPDS scale.

**Results:**

We identified 797 PPD cases, 404 of whom submitted DNA. There were 109 PPP cases, with 66 submitting DNA. PPD cases (86.7% White, mean 4.7 +/− 7.0 years since their episode) came from across Canadian provinces/territories. LCA identified two PPD classes clinically distinct by symptom severity: [1] moderate-severity (mean EPDS = 18.5+/− 2.5; 8.6% with suicidality), and [2] severe (mean EPDS = 24.5+/− 2.1; 52.8% with suicidality).

**Conclusions:**

A mobile application rapidly collected data from individuals with moderate and severe symptoms of PPD, an advantage for genetics where specificity is optimal, as well as from women with a history of PPP, supporting future work using this approach.

**Supplementary Information:**

The online version contains supplementary material available at 10.1186/s12888-022-04363-7.

## Background

Postpartum depression (PPD) affects about 13% of women and is a source of significant illness burden for affected mothers, children, and families [1–3]. Negative outcomes include poor mother-child attachment, delayed child development, and intergenerational transmission of psychiatric disorders [4]. Postpartum psychosis (PPP), the rare but serious counterpart to PPD thought in most cases to represent a severe form of a bipolar mood disorder, occurs in 0.1% of births and can result in infanticide and suicide [5, 6].

Personal and family history of mental disorders are strong risk factors for postpartum mood disorders, suggesting a heritability component [7–9]. Elucidating a genetic basis to these disorders may lead to better risk prediction and disorder-specific treatment. Prior genetic studies have had very small sample sizes, and no studies to date have been sufficiently powered to detect genetic underpinnings of PPD or PPP [10–12]. Rapidly recruiting the large samples of perinatal women required to conduct hypothesis-generating studies that could shed light on the genetics of these conditions is a challenge; new mothers experience competing demands and fear negative responses to disclosure of symptoms, limiting participation in research [13]. Common barriers to recruiting and retaining perinatal women for research include lack of awareness of research, poor access to transportation, and time constraints [14, 15]. As such, digital technologies create new avenues for rapid recruitment due to their increased reach, convenience, and allowance for greater participant anonymity [13, 16]. Reproductive age women are frequent users of online mental health information, making digital recruitment an attractive strategy in this group [17].

The international Postpartum depression Action toward Causes and Treatment (PACT consortium) formed in 2010 with a goal to rapidly collect 100,000 genetic samples worldwide for an international genetics study of postpartum mood disorders [18]. The first set of aims of the consortium were to identify clinical sub-types of PPD that contribute diagnostic heterogeneity, and to elucidate the genetic basis of PPD by conducting the first large genome-wide association study of PPD [19]. In data assembled from a subset of seven of 19 international PACT PPD sites, participants were recruited and data collected using various methods, including in-person and remotely (*n =* 663). A latent class analysis using items from the Edinburgh Postnatal Depression Scale (EPDS), a 10-item scale validated for the identification of PPD in perinatal women, identified five distinct subtypes of PPD, with clear differences in symptom quality [18]. It was expected that these distinct subtypes could be analyzed to determine whether they were associated with specific genetic architecture in future.

The first planned international genetic analysis was a large genome-wide association study (GWAS) of PPD and PPP [20], with the ultimate goal of whole genome sequencing with deep coverage to be performed for the entire sample. However, it was determined that more samples were required. In 2016, the consortium developed and launched an iOS application, the PPD-ACT app, in the United States and Australia, to rapidly identify and collect genetic samples from women with PPD and/or PPP, intending to add as many new countries as possible over time to contribute genetic samples to the international PACT consortium for analysis [18]. In its first year, the US version of the app recruited 7344 women with a history of PPD and biobanked 2946 DNA samples. Women were about a median of 2 years (IQR 1–5 years) since their most severe PPD episode, and had severe illness (EPDS score median 23, IQR: 20–25) [18].

To contribute Canadian data to the PACT consortium, we created a Canadian version of the PPD-ACT iOS app. In the current study, we aimed to evaluate the feasibility of the PPD-ACT iOS app protocol in Canada, including recruitment and data collection, and to identify clinically distinct sub-types of PPD in the sample to contextualize it within the context of other PACT consortium samples. Given that Canada (pop ~ 30 million) has about one-tenth the population of the U. S (pop ~ 300 million),we intended to consider the approach feasible if able to match about 10% of the sample of the US app in its first year, i.e. ~ 734 PPD cases, ~ 295 DNA samples. Based on the US data, we hypothesized that the sample would be weighted toward those with more severe illness.

## Methods

### Study design

The PPD-ACT app was developed using Apple ResearchKit, a software platform developed by Apple Inc. to support researchers using iOS platforms to gather data for medical research purposes. The app was designed by researchers at the University of North Carolina at Chapel Hill in partnership with Little Green Software (now called One Cow Standing: http://www.onecowstanding.com/). We made minor modifications for the Canadian version to meet ethical and regulatory requirements and included Canadian province-specific mental health care resources for women disclosing current symptoms. Women who endorsed suicidality were specifically provided information about a suicide prevention hotline. For the current study, only an English language application was available The study was advertised from April 2017 to June 2019 via social media (e.g., Facebook), institutional websites of the investigators, and word of mouth. Adult Canadian women were invited to download the free PPD-ACT iOS app from the Apple App Store, if they thought they might have experienced PPD or PPP.

### Case identification

Upon downloading the application, PPD was first defined, in lay terms, as follows: “Feeling down or anxious after the birth of a child – the ‘baby blues’ – is pretty common. Sometimes the low mood doesn’t go away and can get intense. This is postpartum depression (PPD), and affects at least 1 woman in every 7 after the birth of a child”. Then, women provided informed consent via self-report via the app for initial phenotypic data collection to determine whether they met criteria for past or current PPD or past PPP. Participating women who met criteria for PPD or PPP based on their answers to the phenotypic data collection, were invited to complete a second informed consent process for DNA sampling, storage, and genetic assays.

We used a modified version of the lifetime Edinburgh Postnatal Depression Scale (EPDS) to detect cases with PPD, asking women to focus on their most severe PPD episode (i.e., current or past) [21]. This version of the EPDS consists of 21 questions - the standard 10 EPDS symptom questions, and then 11 additional questions regarding symptom onset, duration, and level of interference and distress [18]. The EPDS is the most widely used postpartum depression scale, with good reliability, sensitivity, and specificity estimates [22–24], including with online administration [25]. Women were classified as a PPD case if they had (i) EPDS score > 12 after any previous pregnancy [26, 27]; (ii) symptoms lasting 2 weeks or more, with at least mild impairment related to their symptoms; (iii) onset of symptoms in pregnancy or in the first 90 days postpartum (to restrict to perinatal-onset cases); and (iv) no evidence of maternal or child complications that could imply that the symptoms are a result of a stressor such as preterm birth, severe maternal or child illness (to exclude individuals whose symptoms might better be conceptualized as an adjustment disorder). In a previous study using the PPD ACT app, agreement for case status was 99% when women were reassessed using these same criteria 6 months after enrollment [18].

Whether or not a woman met PPD case criteria, she was asked the following 2 questions to identify self-reported PPP: (i) “Postpartum psychosis is rare and happens within the first days to weeks following childbirth. Women may experience strange and unusual beliefs, hear or see things that no one else can, feel very hyper, or do strange things that are very different from their usual behavior. Sleep disturbances are also very common. Do you think postpartum psychosis ever happened to you?” and (ii) “Were you diagnosed with either ‘postpartum psychosis’ or ‘depression with psychotic features’ by a medical professional?”. Those endorsing both questions were asked to submit a DNA sample for future PPP analyses.

Additional variables collected through the app were age (in years), race/ethnicity, province or territory of residence, the number of years since a woman’s worst postpartum mood episode, level of impairment from the symptoms during that episode, and nature of treatment undertaken (any help-seeking, prescription medication use, psychiatric hospitalization).

### Data collection

Phenotypic data were collected directly in the app. The data were encrypted prior to data transfer and stored on secure servers hosted by the UNC, the primary PACT consortium site. A secure web portal hosted by UNC allowed the download of encrypted archives of the Canadian research data for analysis in Canada. No research data were sent to Apple. Participants consenting to genetic data collection were mailed a saliva-based home DNA collection kit (Oragene-DNA kit) with instructions for self-obtaining a sample and returned their sample by pre-paid courier envelope. Up to four follow-up emails were sent to participants whose kit was unreturned after 1 month. Genetic samples were stored in a locked and secure lab in Canada, with GWAS data sent to the international PACT consortium for a future, combined analysis. The genetic material remains in Canada with the PPD-ACT Canada study team as custodians.

### Analyses

The goal of the PACT consortium in creating the app was to rapidly recruit and collect data for a large enough sample for a GWAS as quickly as possible up to 100,000 samples, so there was no set sample size per unit time. However, to assess the feasibility of recruitment with the app in Canada, we calculated the recruitment rate and proportion of eligible participants completing each of phenotypic and genotypic data collection per unit time. Specifically, we recorded the number of participants recruited over the course of the first full year of recruitment, so as to be able to compare this to the recruitment rate of the PPD ACT in the US over its first year, and use the data to determine the value of proceeding to a larger study using the PPD ACT app in Canada. We then described the demographic and clinical characteristics of women with PPD (with or without PPP) and women self-reporting only PPP. EPDS results were reported as a mean score, and for two sub-components: (i) anxiety, using EPDS items 3, 4 and 5 [28]; and (ii) suicidality, defined as responses of *“yes, quite often” or “yes, sometimes”* to EPDS item 10 (“*the thought of harming myself has occurred to me”*).

To determine whether there were clinically distinct subtypes of PPD in the sample, a latent class analysis (LCA) of the PPD cases based on the EPDS symptom profile was then completed. All EPDS items were included, and two, three, and four class solutions were examined. Bayesian Information Criterion (BIC), Akaike’s Information Criterion (AIC), G^2^ and *x*^*2*^ and clinical judgment were considered in final model selection. Subgroups based on class membership were generated. Demographic and clinical characteristics of the subgroups were reported, and compared using chi-square tests and t-tests/analysis of variance. We used RStudio 1.1.456, with the poLCA package 1.4.1.

## Results

### Study recruitment

**Fig. 1:**
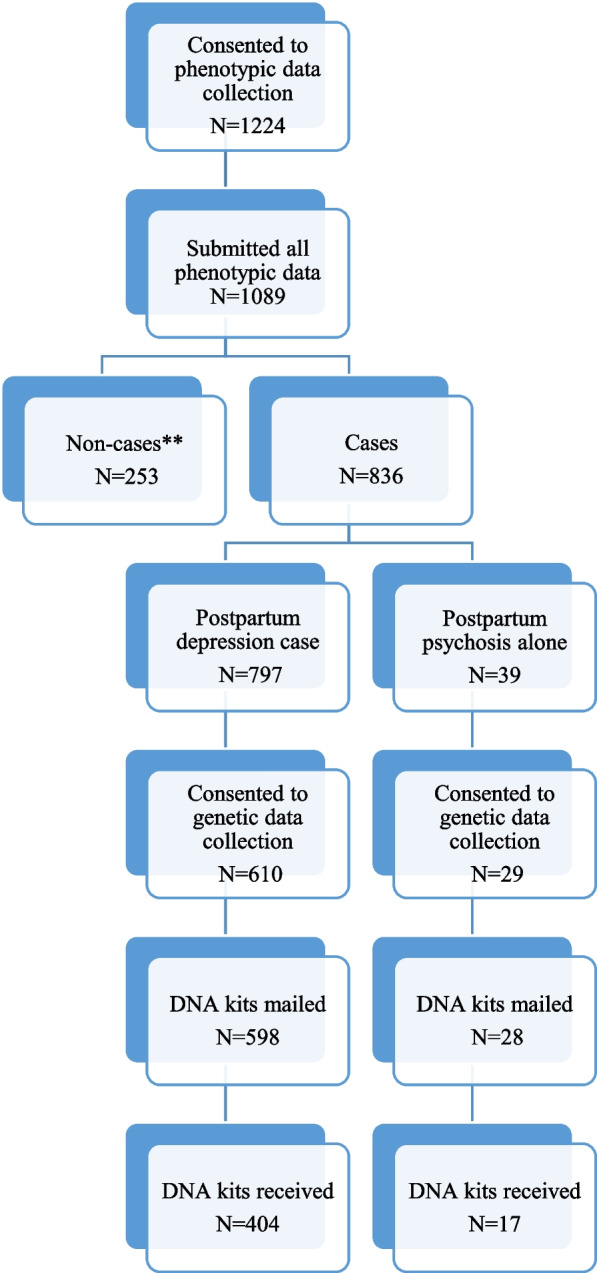
Flow of participant recruitment and data collection. **Excluded due to Edinburgh postnatal depression scale (EPDS) score of less than 13 (*N =* 37), depression symptoms lasing less than 2 weeks (*N =* 22), symptoms with onset outside specified perinatal window (*N =* 137), or other reasons, e.g. bereavement, maternal or child illness (*N =* 57)

1224 women consented to initial participation, of which 1089 (90.0%) provided all information required to assess their case status, with participation from 11 out of 13 of Canada’s provinces and territories (Table S1). From these 1089 participants, 76.8% (*n* = 836) met PPD or PPP case criteria, with the most common reason for not meeting case status being onset of depressive symptoms outside the perinatal window (Fig. 1). Most participants were enrolled in the first year of recruitment (*n =* 774, 63%), meeting the recruitment target for the pilot. Most PPD cases (*n =* 598 out of 797, 75.0%) agreed to the genetic component of the study, with 404 (67.6%) submitting a sample. Of 108 women reporting PPP, 39 were not also a PPD case; 17 out of these 39 (60.7%) submitted DNA. The average time to sample return was 33.6 days (SD 37.7).

PPD cases were predominantly White (86.7%), with mean age of 34.8 years (SD 7.9) (Table 1). Mean number of years since the index PPD episode was 4.7 (SD 7.0) and 19.3% (*n* = 154) were reporting current symptoms. About 47.7% (*n* = 380) reported symptom onset in the first 4 weeks after delivery. Mean EPDS score was 22.3 (SD 3.8), 54.7% reported that their symptoms *often* interfered with their ability to function, and 37.4% reported suicidal thoughts during the episode. Most (69.3%) had sought professional help, with 49.3% using prescription medication and 6.3% hospitalized. Women with PPP had very high EPDS scores (mean 23.1, SD 4.5). About 66.7% reported depressive symptoms *often* interfering with function, and 54.0% reported suicidality during the episode. Almost three-quarters (74.4%) reported prescription medication use, and about one-quarter (25.6%) were hospitalized during their episode (Table 1).

**Table 1:** Sociodemographic and clinical characteristics of 797 women with postpartum depression (PPD) and 39 women with a history of postpartum psychosis (PPP), N(%) unless otherwise specified

Variable	PPD (*n =* 797)	PPP alone (*n =* 39)
Demographics		
Mean age (SD) in years	34.8 (7.9)	34.5 (8.8)
Race/Ethnicity^a^		
White	691 (86.7)	33 (84.6)
Asian	40 (5.0)	4 (10.3)
Indigenous	23 (2.9)	< 5
Black	8 (1.2)	< 5
Hispanic	8 (1.2)	< 5
Other	23 (2.0)	< 5
Time (SD) in years since index episode	4.7 (7.0)	5.7 (4.5)
Episode occurred in the past year	154 (19.3)	6 (15.4)
Clinical and Health Service Use Characteristics		
Mean (SD) Edinburgh postnatal depression scale (EPDS)	22.3 (3.8)	23.1 (4.5)
Mean (SD) EPDS Anxiety Subscale^b^	7.9 (1.4)	8.0 (1.5)
EPDS Suicidality^c^	298 (37.4)	21 (54.0)
Self-reported physician diagnosis of psychosis	108 (13.6)	39 (100)
Sought professional help for mental illness	552 (69.3)	35 (89.7)
Hospitalized for mental illness	50 (6.3)	10 (25.6)
Received medication for mental illness	393 (49.3)	29 (74.4)
PPD Characteristics		
Onset of depressive symptoms		
During first trimester of pregnancy	119 (14.9)	< 5
During second trimester of pregnancy	73 (9.2)	< 5
During third trimester of pregnancy	75 (9.4)	< 5
0–4 weeks after delivery of pregnancy	380 (47.7)	6 (15.4)
1–3 months after delivery of pregnancy	150 (18.8)	< 5
Length of depressive symptoms		
Less than 2 weeks	–	< 5
2–4 weeks	76 (9.5)	< 5
1–3 months	137 (17.2)	7 (17.9)
3 or more months	584 (71.8)	28 (71.8)
Interference of depressive symptoms		
Never or rarely	4 (0.5)	0 (0)
Rarely	58 (7.3)	4 (10.3)
Sometimes	299 (37.5)	9 (23.1)
Often	436 (54.7)	26 (66.7)

^**a**^Missing *n =* 4 in PPD cases. Cells with < 5 suppressed for anonymity. ^**b**^EPDS anxiety subscale calculated from items 3–5, (3:*“I have blamed myself unnecessarily when things went wrong”*), (4:*“I have been anxious or worried for no good reason”*), and (5:*“I have felt scared or panicky for no very good reason”*) ^**c**^Responses of *sometimes* or *often* on EPDS item 10 (‘*the thought of harming myself has occurred to me’)*

### Latent class analysis

Fit indices improved from one to two classes but only marginally thereafter (Table 2). There were no clinically important distinctions when moving to solutions with three and four classes, so the 2-class solution was selected, with about 35% of women predicted to fall into Class 1, and ~ 65% into Class 2. Differences in symptoms between classes were apparent (Table 3). For both classes, there was a high probability of the highest possible (most severe) score on the EPDS item “I have been anxious for no good reason”(yes, very often) and the second most severe score on “ability to laugh” (definitely not so much now). However, Class 1 had high probabilities of scores of 1 or 0 (i.e. lower severity) for the other items. In contrast, Class 2 had high probability of the second most severe score for “looking forward with enjoyment to things” (definitely less than I used to)” and “I have been so unhappy that I have had difficulty sleeping” (yes, sometimes). Class 2 also had a high probability of the most severe response to “the thought of harming myself has occurred to me” (Yes, quite often). As such, we considered Class 1 to be a “moderate-severity subgroup” and Class 2 to be a “severe” subgroup.

**Table 2:** Best fit indices and predicted probability for the Latent Class Analysis of postpartum depression (PPD) cases

	Indicator of Fit				Predicted Probability of Membership			
Number of latent classes	BIC	AIC	G^2^	X^2^	Class 1	Class 2	Class 3	Class 4
1	14,505.73	14,365.31	4749.673	7,553,077	0.3488	0.6512	–	–
2	13,739.31	13,453.78	3776.140	10,736,478	0.3689	0.3513	0.2798	–
3	13,704.63	13,273.99	3534.357	8,637,221	0.3036	0.3212	0.1593	0.2158
4	13,703.08	13,127.34	3325.701	3,295,150	0.2961	0.3074	0.1619	0.0853

*BIC* Bayesian Information Criteria, *AIC* Akakie Information Criteria

**Table 3:** Associations between scores on each EPDS item, and the latent classes (2-class solution) for the 797 women with PPD included in the latent class analysis, presented as probability (p) of achieving the stated score on a given item for each class*

Item	Class	Item Responses (Left to Right Increasing in Severity)			
1. I have been able to laugh and see the funny side of things		As much as I always could	Not quite so much now	Definitely not so much now	Not at all
	**Class 1**	0.0808	**0.5476**	**0.3435**	0.0281
	**Class 2**	0.0108	0.1241	**0.6188**	0.2463
2. I have looked forward with enjoyment to things		As much as I ever did	Rather less than I used to	Definitely less than I used to	Hardly at all
	**Class 1**	0.1290	**0.6384**	0.2326	0.0000
	**Class 2**	0.0096	0.2308	**0.6190**	0.1407
3. I have blamed myself unnecessarily when things went wrong		No, never	Not very often	Yes, some of the time	Yes, most of the time
	**Class 1**	**0.8162**	0.1649	0.0090	0.0099
	**Class 2**	**0.5777**	**0.3042**	0.0976	0.0205
4. I have been anxious or worried for no good reason		No, not at all	Hardly ever	Yes, sometimes	Yes, very often
	**Class 1**	0.0000	0.0025	0.0804	**0.9171**
	**Class 2**	0.0069	0.0883	0.2380	**0.6668**
5. I have felt scared or panicky for no very good reason		No, not at all	No, not much	Yes, sometimes	Yes, quite a lot
	**Class 1**	**0.6913**	0.2530	0.0455	0.0101
	**Class 2**	**0.3963**	**0.3469**	0.2332	0.0236
6. Things have been getting on top of me		No, I have been coping as well as ever	No, most of the time I have coped quite well	Yes, sometimes I haven’t been coping as well as usual	Yes, most of the time I haven’t been able to cope
	**Class 1**	**0.9693**	0.0287	0.0000	0.002
	**Class 2**	**0.7822**	0.1973	0.0206	0.000
7. I have been so unhappy that I have had difficulty sleeping		No, not at all	Not very often	Yes, sometimes	Yes, most of the time
	**Class 1**	**0.6478**	0.2543	0.0924	0.0055
	**Class 2**	0.2214	**0.3446**	**0.3441**	0.0900
8. I have felt sad or miserable		No, not at all	Not very often	Yes, quite often	Yes, most of the time
	**Class 1**	**0.8985**	0.1015	0.0000	0.0000
	**Class 2**	0.2841	**0.5856**	0.1132	0.0172
9. I have been so unhappy that I have been crying		No, never	Only occasionally	Yes, quite often	Yes, most of the time
	**Class 1**	**0.8102**	0.1756	0.0122	0.0020
	**Class 2**	0.2965	**0.4915**	0.1949	0.0171
10. The thought of harming myself has occurred to me		Never	Hardly ever	Sometimes	Yes, quite often
	**Class 1**	0.1523	**0.3830**	0.2315	0.2332
	**Class 2**	0.0104	0.0836	0.2572	**0.6488**

*p > 0.30 are considered a strong association, and are bolded in the above table

Upon assigning the 797 women with PPD in the sample to either Class 1 (*n* = 278) or Class 2 (*n* = 519), there were no differences between the subgroups with respect to age, or race or ethnicity (Table 4). Those in Class 2 reported a longer time since the episode had occurred. The clinical characteristics of the women assigned to the classes were consistent with the “moderate-severity” and “severe” naming conventions. For women assigned to Class 1, the EPDS mean score (18.0, SD 2.5) was in the moderate range, with 8.6% (*n* = 24) reporting suicidal thoughts and 43.1% reporting a duration of illness of 3 months or less. For women assigned to Class 2 (*n* = 519), the mean EPDS score was in the severe range (24.5, SD 2.1), 52.8% reported suicidal thoughts, and 82.1% reported illness duration of 3 months or longer. Interference with quality of life, prescription medication use and psychiatric hospitalization during the episode was also more common in this subgroup.

**Table 4:** Sociodemographic and clinical characteristics of 797 women with postpartum depression (PPD) by latent class, presented as N(%) unless otherwise specified

Variable	Class 1 Moderate-Severity (*n =* 278)	Class 2 Severe (*n =* 519)	*P* value
Demographics			
Mean age (SD) in years	30.5 (4.5)	29.9 (4.8)	0.24
Race/Ethnicity^a^			0.56
Indigenous	5 (1.8)	18 (3.5)	
Asian	14 (5.0)	26 (5.0)	
White	244 (87.8)	447 (86.1)	
Other	12 (4.3)	27 (5.2)	
Time (SD) in years since index episode	3.9 (6.6)	5.2 (7.2)	0.009
Clinical and Health Service Use Characteristics			
Mean (SD) Edinburgh postnatal depression scale (EPDS)	18.0 (2.5)	24.5 (2.1)	< 0.001
Mean (SD) EPDS Anxiety Subscale^b^	7 (1.7)	9 (0.96)	< 0.001
EPDS Suicidality^c^	24 (8.6)	274 (52.8)	< 0.001
Self-reported physician diagnosis of psychosis	20 (7.2)	89 (17.1)	< 0.001
Sought professional help for mental illness	142 (51.1)	410 (79.0)	< 0.001
Hospitalized for mental illness	6 (2.2)	44 (8.5)	< 0.001
Received medication for mental illness	86 (30.9)	307 (59.2)	< 0.001
PPD Characteristics			
Onset of depressive symptoms			0.002
During first trimester of pregnancy	46 (16.5)	73 (14.1)	
During second trimester of pregnancy	19 (6.8)	54 (10.4)	
During third trimester of pregnancy	27 (9.7)	48 (9.2)	
0–4 weeks after delivery of pregnancy	115 (41.4)	265 (51.1)	
1–3 months after delivery of pregnancy	71 (25.5)	79 (15.2)	
Length of depressive symptoms			< 0.001
2–4 weeks	46 (16.5)	30 (5.8)	
1–3 months	74 (26.6)	63 (12.1)	
3 or more months	158 (56.8)	426 (82.1)	
Interference of depression symptoms			< 0.001
Never or rarely	4 (1.4)	0 (0)	
Rarely	52 (18.7)	6 (1.2)	
Sometimes	151 (54.3)	148 (28.5)	
Often	71 (25.5)	365 (70.3)	

^a^Missing *n =* 3 in Class 1, *n =* 1 in Class 2 ^b^EPDS anxiety subscale calculated from items 3–5, (3:*“I have blamed myself unnecessarily when things went wrong”*), (4:*“I have been anxious or worried for no good reason”*), and (5:*“I have felt scared or panicky for no very good reason”*) ^c^Responses of *sometimes* or *often* on EPDS item 10 (‘*the thought of harming myself has occurred to me’)*

## Discussion

This study offers a description of a novel method to recruit large samples for genetic research in a very difficult-to-recruit population: postpartum women. Given the need to understand the feasibility of digital platforms to collect psychiatric genetic information [29], this study adds foundational information to the literature base. We showed that the app-based data collection appears to be a rapid and acceptable approach for a population that is often difficult to engage in research due to their many competing priorities [13]. The PPD-ACT mobile app enrolled over 1000 Canadian women who suspected they might have experienced PPD or PPP, and over 400 genetic samples were collected from women with “moderate-severity” and “severe” PPD, as well as from women self-reporting PPP, providing evidence for the PPD-ACT app as a highly feasible method for genotypic PPD data collection in Canada.

The results of our LCA suggest that the mobile app recruited women of two clinically-distinct subtypes – one which we termed to be “moderate-severity” (mean EPDS score 18/30), and one on the “severe” end of the PPD symptom spectrum (mean EPDS score extremely high at 24.5/30). Both subtypes were linked to high anxiety, but in the “severe” subgroup, there was significant anhedonia (lack of enjoyment) and suicidality. Previous LCAs from clinical studies of PPD have identified three to five clinically distinct subgroups of women [30], also distinguished by the severity of symptoms, usually with low, moderate, and high symptom severity groups. In prior data PACT PPD sites (*n =* 663), an LCA using the EPDS items identified five distinct subtypes of PPD: severe anxious depression, moderate anxious depression, anxious anhedonia, pure anhedonia, and resolved depression [30]. While we did not have a clinically distinct “anhedonia” subgroup in our data, anxiety and anhedonia were the main symptom distinguishers in our sample as well. Our findings that women recruited by the app had fairly severe PPD was also consistent the US PPD-ACT sample, where no LCA was conducted but the median EPDS score was 23 (IQR 20–25) [18], very similar to our mean EPDS score of 22.3 (SD 3.8). The criteria used in our study and that of the US PPD-ACT study to try to increase diagnostic accuracy – i.e., ensuring at least 2 weeks of symptoms, excluding women with non-perinatal onset and other potential explanations for symptoms (e.g. maternal or child illness) may explain our higher-severity recruitment. Women with more severe illness (past or present) may also be differentially motivated to participate in a study on elucidating a genetic basis for the disorder. Regardless, a specific sample (i.e., versus a more sensitive sample with high likelihood of false positive inclusion) is preferred for genetic analyses.

When considering a larger scale study in Canada, to improve accessibility and inclusivity, additional platforms are likely warranted. Web-based participation is an option, and Android devices are less costly to the user making future expansion of the platform to Android a logical next step; a US Android version has been implemented. Further, the biggest regional gaps in study recruitment were in Quebec and New Brunswick; this may be improved with a French translation. Finally, while the study was not focused on identifying individuals for clinical care, almost one in five PPD cases were reporting current symptoms. Thus, in future, the app may be leveraged to address the challenge of low treatment rates for PPD [31] as a novel way to engage and direct women to treatment who might be otherwise reticent about disclosing symptoms to their healthcare providers or not know where to receive help.

## Conclusions

Given the large burden of PPD on women and families, even small improvements in treatment could result in a large public health impact. The PPD-ACT mobile app is a feasible method of collecting genotypic information to accelerate knowledge about the etiology of PPD and drive new discoveries with respect to predictive biomarkers and treatment. It may also be a useful strategy to identify women and direct them to appropriate services. Next steps for a larger study will be expansion to different languages, adapting the Android version for use in Canada, and enabling web-based participation for women without mobile devices, to improve generalizability of ensuant findings to diverse groups of women.

## Supplementary Information

**Additional file 1: Supplemental Table 1.** Geographic breakdown compared to 2016 census data, in N(%). *Missing *n =* 152 from case groups. **Missing *n =* 175 from non-cases. Note: Expected percentage calculated with an overall Canadian population of 35,151,728.

## Data Availability

Data are available from the corresponding authors upon reasonable request; research ethics board permission was not received for public posting of the data in repositories.

## Abbreviations

PPD: Postpartum depression
PPP: postpartum psychosis
GWAS: genome-wide association study
EPDS: Edinburgh postnatal depression scale
LCA: latent class analysis
BIC: Bayesian Information Criterion
AIC: Akaike’s Information Criterion
