# Supplementary material for: The PPD-ACT app in Canada: feasibility and a latent class analysis of participants with postpartum depression recruited to a psychiatric genetics study using a mobile application
Source: BMC Psychiatry. 2022 Nov 24;22:735. doi: 10.1186/s12888-022-04363-7 (PMC9700884; doi:10.1186/s12888-022-04363-7)
Supplement: Supplementary file 1 — Additional file 1: Supplemental Table 1. Geographic breakdown compared to 2016 census data, in N(%). *Missing n = 152 from case groups. **Missing n = 175 from non-cases. Note: Expected percentage calculated with an overall Canadian population of 35,151,728. [file 12888_2022_4363_MOESM1_ESM.docx]

**Supplemental Table 1. Geographic breakdown compared to 2016 census data, in N(%)**

| **Province or territory** | **Prevalence of case group** | **Prevalence of non-cases**** | **Expected population** |
| --- | --- | --- | --- |
| Alberta | 63 (9.2) | 7 (9.0) | 4,067,175 (11.6) |
| British Columbia | 89 (13.0) | 8 (10.3) | 4,648,055 (13.2) |
| Manitoba | 29 (4.2) | 1 (1.3) | 1,278,365 (3.6) |
| New Brunswick | 3 (0.4) | 2 (2.6) | 747,101 (2.1) |
| Newfoundland & Labrador | 6 (0.9) | - | 519,716 (1.5) |
| Northwest Territories | 0 (0) | - | 41,786 (0.1) |
| Nova Scotia | 17 (2.5) | 2 (2.6) | 923,598 (2.6) |
| Nunavut | 0 (0) |  | 35,944 (0.1) |
| Ontario | 416 (60.8) | 46 (59.0) | 13,448,494 (38.3) |
| Prince Edward Island | 3 (0.4) | - | 142,907 (0.4) |
| Quebec | 23 (3.4) | 5 (6.4) | 8,164,361 (23.2) |
| Saskatchewan | 33 (4.8) | 7 (9.0) | 1,098,352 (3.1) |
| Yukon Territory | 2 (0.3) | - | 35,874 (0.1) |

*Missing *n=*152 from case groups

**Missing *n=*175 from non-cases

Note: Expected percentage calculated with an overall Canadian population of 35,151,728
